# Results of a European interlaboratory comparison on gross alpha/beta activity determination in drinking water

**DOI:** 10.1007/s10967-015-3955-2

**Published:** 2015-02-19

**Authors:** Viktor Jobbágy, Jana Merešová, Edmond Dupuis, Pieter Kwakman, Timotheos Altzitzoglou, Andrej Rožkov, Mikael Hult, Håkan Emteborg, Uwe Wätjen

**Affiliations:** 1 Joint Research Centre, Institute for Reference Materials and Measurements (JRC-IRMM), European Commission, Retieseweg 111, 2440 Geel, Belgium; 2 Low Level Radioactivity Measurements Expert Group, Institute for Environment, Health and Safety, SCK•CEN, Boeretang 200, 2400 Mol, Belgium; 3 Laboratory for Radiation Research, RIVM, PO Box 1, 3720 BA Bilthoven, The Netherlands; 4 Present Address: Kievermondeveld 74, 2440 Geel, Belgium; 5 Present Address: Waste and Disposal Expert Group, Institute for Environment, Health and Safety, SCK•CEN, Boeretang 200, 2400 Mol, Belgium

**Keywords:** Interlaboratory comparison, Gross alpha/beta activity, Drinking water, Environmental radioactivity

## Abstract

An interlaboratory comparison was organised by JRC-IRMM among environmental radioactivity monitoring laboratories for the determination of gross alpha/beta activity concentration in drinking water. Independent standard methods were used for the reference value determination. The performance of participating laboratories was evaluated with respect to the reference values using relative deviations. Sample preparation and measurement methods used by the participating laboratories are detailed, in particular in the view of method-dependency of the results. Many of the participants’ results deviate by more than two orders of magnitude from the reference values regardless of the techniques used. This suggests that gross methods need revision.

## Introduction

Gross alpha/beta activity measurements are widely applied as a screening technique in many fields (e.g. environmental monitoring, industrial applications). Water intended for drinking purposes has to be analysed first for gross alpha/beta activity according to national and international standards and recommendations [1]. Anticipating the new EU drinking water directive [2], which includes gross alpha/beta activity screening levels, JRC-IRMM organised in 2012 an interlaboratory comparison (ILC) to check the fitness for purpose of this method and the performance of European monitoring laboratories.

The EU member state national representatives (in the Art. 35–36 expert group) under the Euratom Treaty [3] nominated the participants. IRMM then contacted the nominated laboratories and distributed the water samples. The participants submitted signed reports including their results together with their answers to a questionnaire. The ILC was evaluated on the basis of the measurement values that the 71 participant laboratories reported.

## Sample selection and treatment

Several waters of natural origin were analysed to find representative samples for this ILC. To make the final selection, the activity concentration of the alpha-emitting radionuclides, salinity, chemical composition, directives and recommendations were taken into account. Three water samples (Water A, Water B: two commercially available natural mineral waters; Water C: one laboratory-made spiked water) were selected. Details on the preliminary radioanalytical characterization and selection are published elsewhere [4].

Water B was bottled into anonymous 1.5 L polyethylene terephthalate bottles by a mineral water producer company. Water A and Water C were prepared as follows.

Water B was produced from a commercial mineral water from France. Two perfluoroalkoxy polymers (PFA)-lined drums of 550 L were filled with the mineral water and the water was thereafter re-circulated for 24 h at 15 L/min using two inert Iwaki bellow pumps. During filling an intermediate polycarbonate buffer tank of 20 L (Nalgene, Rochester, NY, USA) was used and the water was pumped simultaneously from the two tanks into the buffer tank. The buffer tank was placed in a clean bench and the water bottles were filled manually by opening and closing the tap of the buffer tank. Prior to filling, the buffer tank was rinsed with 2 × 10 L of Type 1 water (18.2 MΩ cm, 0.056 µS/cm at 25 °C and TOC < 5 ng/mL from a Milli-Q Advantage system (Millipore, Billerica, MA, USA) and 20 L of mineral water. In this manner 777 bottles were filled. The 1-L bottles were made of high density polypropylene (HDPE) with a leak-proof HDPE-screw cap (Nalgene).

Water C was a spiked Type 2 water from a Millipore ELIX-35 system (>5 MΩ cm, 0.2 µS/cm at 25 °C and TOC < 30 ng/mL) with added inorganic salt mixture. During several days 500 L of Type 2 water was collected in portions into one PFA-lined drum of 550 L. Subsequently 120 g of salt mixture of CaCl_2_ and Sr(NO_3_)_2_ was added. Thereafter 2 L of concentrated nitric acid was added (pH = 1.2 ± 0.1) followed by ^90^Sr/^90^Y and ^241^Am spike. The contents were thereafter mixed using the Iwaki inert bellows pump of the water handling system for 16 h at 15 L/min. Subsequently 482 of the 1-L HDPE bottles (Nalgene) were filled as described above. Samples were stored in a dark and dry place at room temperature.

## Reference value determination

The reference value determination was done in three independent laboratories where the four most common routine methods were used (Table 1).

**Table 1:** Methods used for the determination of gross alpha/beta reference values

Collaborator	Method
Belgian Nuclear Research Centre (SCK•CEN), Belgium	Evaporation, solid scintillation counting (ISO 10704)
The National Institute for Public Health and the Environment (RIVM), The Netherlands	Spike addition, evaporation and gas flow proportional counting (ISO 9696/9697)
Institute for Reference Materials and Measurements (EC-JRC-IRMM), Belgium	Co-precipitation, gas flow proportional counting (ISO 10704) and ^40^K activity concentration determination by gamma-ray spectrometry^a^
	Thermal pre-treatment, liquid scintillation counting (ISO 11704)

^a^Complimentary method for ISO 10704 co-precipitation approach

The reason for using a complimentary method for the ISO 10704 co-precipitation approach was that ^40^K activity was not included in the gross beta activity results. During the sample preparation ^40^K was precipitated neither as sulphate nor as hydroxide, but it stayed in the solution and eventually the analysts using ISO 10704 co-precipitation approach would fail to detect it.

The ^40^K activity concentration determination was performed for the natural waters (Water A and B). These samples were measured on the HPGe-detector Ge-4 in the underground laboratory HADES using ultra low-level gamma-ray spectrometry (ULGS) [5, 6].

The homogeneity of the samples within the whole batch and the short term stability during the ILC campaign were checked. The homogeneity of the radionuclides in the matrix was evaluated using the SoftCRM version 2.0.10 software following the certification principles for reference materials as given in ISO/IEC Guide 35 [7].

For the homogeneity study a random stratified method was used to avoid systematic errors within the batch. From each batch of water eight to ten bottles were randomly selected and analysed using gross measurements and radionuclide specific analysis of the natural origin alpha emitting radionuclides (Water A and B). This included the activity concentration determination of the main contributing alpha-emitting radionuclides to the gross alpha activity concentration (e.g. in case of Water B the activity concentration of ^226^Ra was determined). The scatter of the results from the gross measurements was larger than that from the homogeneity values from the radionuclide specific analysis, so the former were used in the uncertainty budget.

In the case of bottled waters, the main contribution to their instability was the adsorption of radionuclides to the container wall. Short term stability analysis was done on a monthly basis using gross measurements and the aforementioned radionuclide specific analysis (Water A and B). The first stability measurements were done already 2 months before the beginning of the ILC and the last 1 month after the submission of the last result.

The contribution of uncertainties from the characterization of the material (*u*_char_), homogeneity between bottles (*u*_bb_), and the short term stability (*u*_sts_,) are presented in Table 2.

**Table 2:** Contribution of uncertainties to the expanded uncertainty of the reference values (%)

Sample	*u*_char_	*u*_bb_	*u*_sts_
Gross alpha activity			
Water A	19.6	13.1	4.8
Water B	6.0	1.5	2.0
Water C	7.5	3.4	6.1
Gross beta activity			
Water A	6.0	2.0	5.9
Water B	8.5	1.5	2.0
Water C	7.4	2.8	4.7

As shown, the largest part of the uncertainty comes from the characterization followed by the short term stability, except in the case of the gross alpha activity in Water A, where the uncertainty contribution from homogeneity is much higher than that from the short term stability.

The combined standard uncertainty of the mean of the measurement results from the reference measurements and is calculated in Eq. (1).

1 $$ u_{\text{char}} = \frac{{\sqrt {\sum\nolimits_{i = 1}^{n} {(u_{c,i} )^{2} } } }}{n} $$

where:

- *u*_c*,i*_ is the combined standard uncertainty of the laboratory or method result [8], and
- *n* is the number of laboratories considered.

The expanded uncertainty (*U*_ref_) of the reference value is calculated by using Eq. (2).

2 $$ u_{\text{ref}} = k \times \sqrt {u_{\text{char}}^{2} + u_{\text{bb}}^{2} } + u_{\text{sts}}^{2} $$

where:

- *k* is the coverage factor (*k* = 2) at ~95 % confidence interval,
- *u*_char_ is the combined standard uncertainty of the mean of the measurement results from the laboratories contributing to the reference value,
- *u*_bb_ is the uncertainty of the activity concentration between bottles of the same batch, and
- *u*_sts_ is the uncertainty due to the short-term stability of the samples (longer than the duration of the comparison exercise).

Table 3 gives the reference values for the three waters analysed and used for the ILC, with their expanded uncertainties.

**Table 3:** Reference activity concentration values (*A*_ref_) of the three waters used in the ILC and their expanded uncertainties (*U*_ref_) (coverage factor *k* = 2)

Parameter	Reference values with expanded uncertainty (*A*_ref_ ± *U*_ref_; mBq L^−1^)		
	Water A	Water B	Water C
Gross alpha activity	47.5 ± 22.8	434.7 ± 56.6	954.5 ± 77.3
Gross beta activity	309.8 ± 57.4	190.4 ± 32.6	1037.3 ± 83.0

## Evaluation of ILC results

The evaluation of the participant’s results is based on their per cent difference or relative deviation from the reference value Eq. (3) [9].

3 $$ D_{\% } = 100 \times \frac{{A_{\text{lab}} - A_{\text{ref}} }}{{A_{\text{ref}} }} $$

where:

- *A*_lab_ is the participant laboratory result (mean activity concentration),
- *A*_ref_ is the reference value.

The results are considered compatible if they fall within the ±30 % range from the reference value and incompatible if they fall outside this range. The 30 % criterion is arbitrary, based on the estimation that the analysis is feasible within this level of deviation.

## Methods used by ILC participants

Besides sending the measurement results, laboratories submitted answers to a questionnaire giving details of their laboratory and routine procedures. From the questionnaire it turned out that 65 laboratories work according to a quality system (mainly ISO 9000 and ISO 17025) and 58 laboratories are either accredited, authorized, certified or have a combination of these three. In 65 laboratories, the same routine analytical procedure was used for the ILC samples as for their regular routine samples.

The amount of water used for the preparation of a single measurement sample ranged from 5 mL up to 5 L. Details on the sample preparation and measurement techniques are presented in Table 4. The measurement time ranged from 1,800 s to 3 days. For the counting efficiency calibration the following radionuclides were used: ^241^Am, U_nat_, ^239^Pu, ^226^Ra, ^210^Po, ^236^U for alpha; and ^40^K, ^90^Sr/^90^Y, ^36^Cl, ^137^Cs, ^210^Pb, ^14^C, ^3^H for beta. These radionuclides cover a wide alpha/beta energy range (18.6–1175.6 keV). Furthermore, one laboratory reported to use ^226^Ra for beta calibration.

**Table 4:** Number of laboratories for sample preparation and measurement techniques used for determining the gross activities

	Number of laboratories
Sample preparation method	
Evaporation to complete dryness	36
Evaporation and mixing with LSC cocktail	16
Evaporation to complete dryness, Coprecipitation	7
Other^a^	4
Coprecipitation	3
Evaporation to complete dryness, Other^a^	3
Measurement technique	
Proportional counter	42
Liquid scintillation counter	22
Scintillation counter (solid)	10
Semiconductor Si detector	2
i-Matic Si-det	1
Grid ionization chamber	1

^a^Category “other” not specified by the participants

The most used sample preparation method was evaporation to dryness with no further sample treatment. The second most used method was by evaporation (thermal pre-concentration) of an aliquot of the sample to a smaller volume and by mixing it with LSC cocktail. Coprecipitation was applied in ten cases and other techniques were used by seven laboratories.

Among the 49 participants who used other techniques than liquid scintillation counting (LSC), 20 laboratories answered yes and 29 no to the question if they have a procedure for hygroscopic residue. These 49 laboratories deposit the residue onto the planchet in many different ways as listed, like automatic evaporation, residue homogenization with a solvent, evaporation of the last few mL on the planchet, direct evaporation on filter paper, direct evaporation and mechanical homogenization.

The most popular measurement techniques were proportional counting, LSC and solid state scintillation counting. Few laboratories applied some non-conventional gross counting like semiconductor Si detector, i-Matic Si-det and grid ionization chamber.

In the case of LSC, the following sample to cocktail ratios were used: 1:4, 2:3, 1:21, 1:3 and 2:1. Only five out of the 21 laboratories using LSC applied quench correction. The type of LSC vials used were: polyethylene (used by 10 laboratories), Teflon coated (9), low potassium glass (1), glass (1) and other (1). One of the laboratories used two different vials. The procedures for the determination of background used by the participant laboratories are summarized in Table 5.

**Table 5:** Procedures used for gross alpha/beta background determination by the participants

Background determination procedure	Number of laboratories
Empty planchette	35
Blank samples	7
Acidified water + LS cocktail	5
Distilled water + LS cocktail	5
ZnS(Ag) powder	3
Background sample in nearly the same chemical composition as the water sample	2
CaSO_4_ spread on planchet	1
Filter paper on a planchet	1
Acidified water + Radon removal + LS cocktail	1
No definite answer	11

As seen, there are nine different approaches for the background determination which may be a reason for biased results. Moreover, there were 11 laboratories that did not provide definite answers but we assume that they might have used one of the nine background determination approaches. Comparing the gross alpha/beta detection limits with the detection limits given in the new drinking water directive (Table 6) one can see there are laboratories not complying with the requirements.

**Table 6:** Limit of detection of gross alpha/beta activity concentrations reported by the participant laboratories in mBq L^−1^

Gross alpha	Gross beta
Limit of detection reported by the participants (mBq L^−1^)	
1.4–340	0–424
Limit of detection (mBq L^−1^) from the new drinking water directive [2]	
40	400

## Results of the ILC

The 71 registered participant laboratories were requested to determine the gross alpha and beta activity concentration of three different water samples. This means that each participant could submit maximum six independent measurement results with their corresponding expanded uncertainties.

**Fig. 1:**
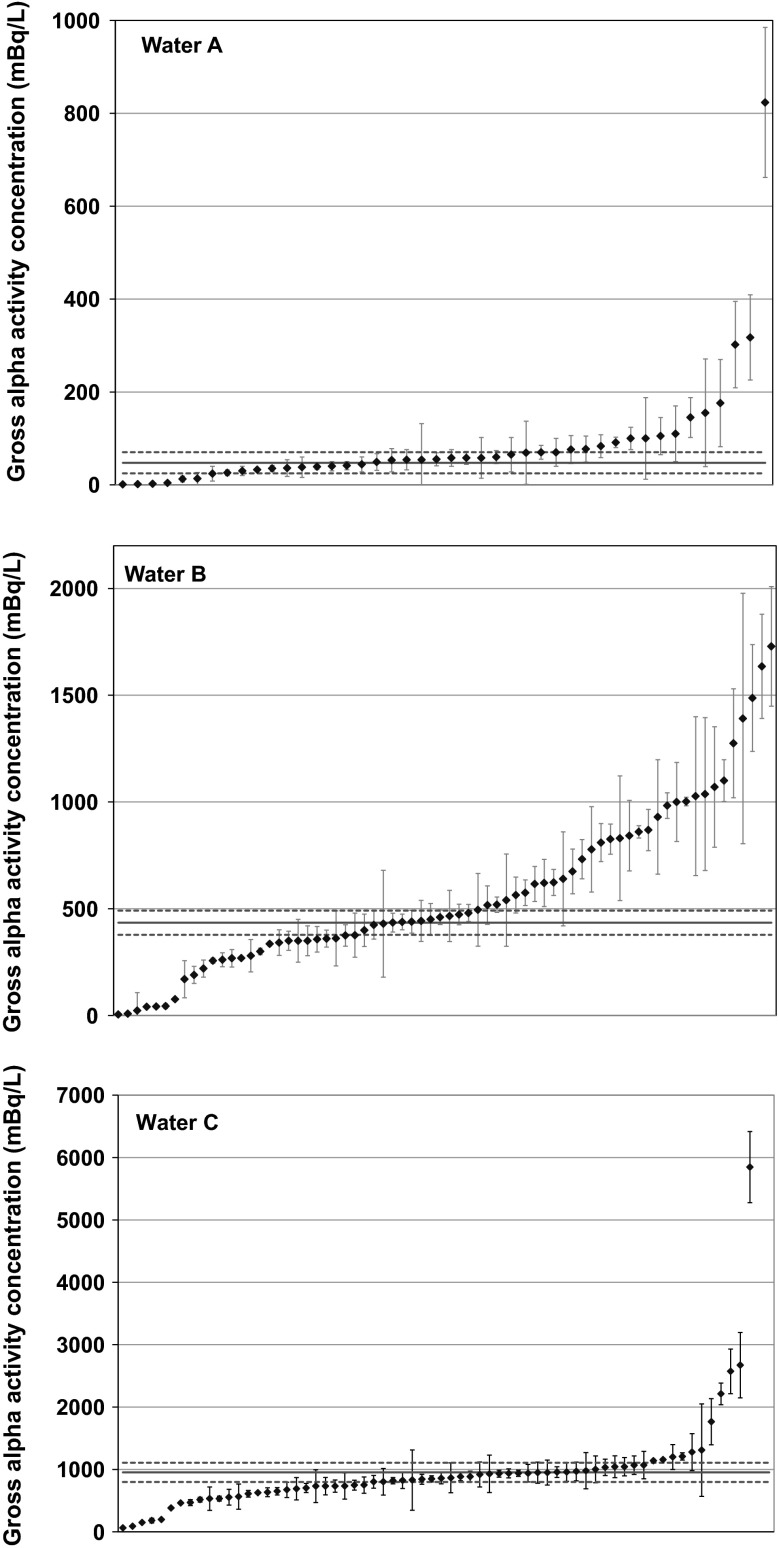
Results of gross alpha activity concentration sorted in ascending order. *Error bars* represent expanded uncertainties (*k* = 2)

**Fig. 2:**
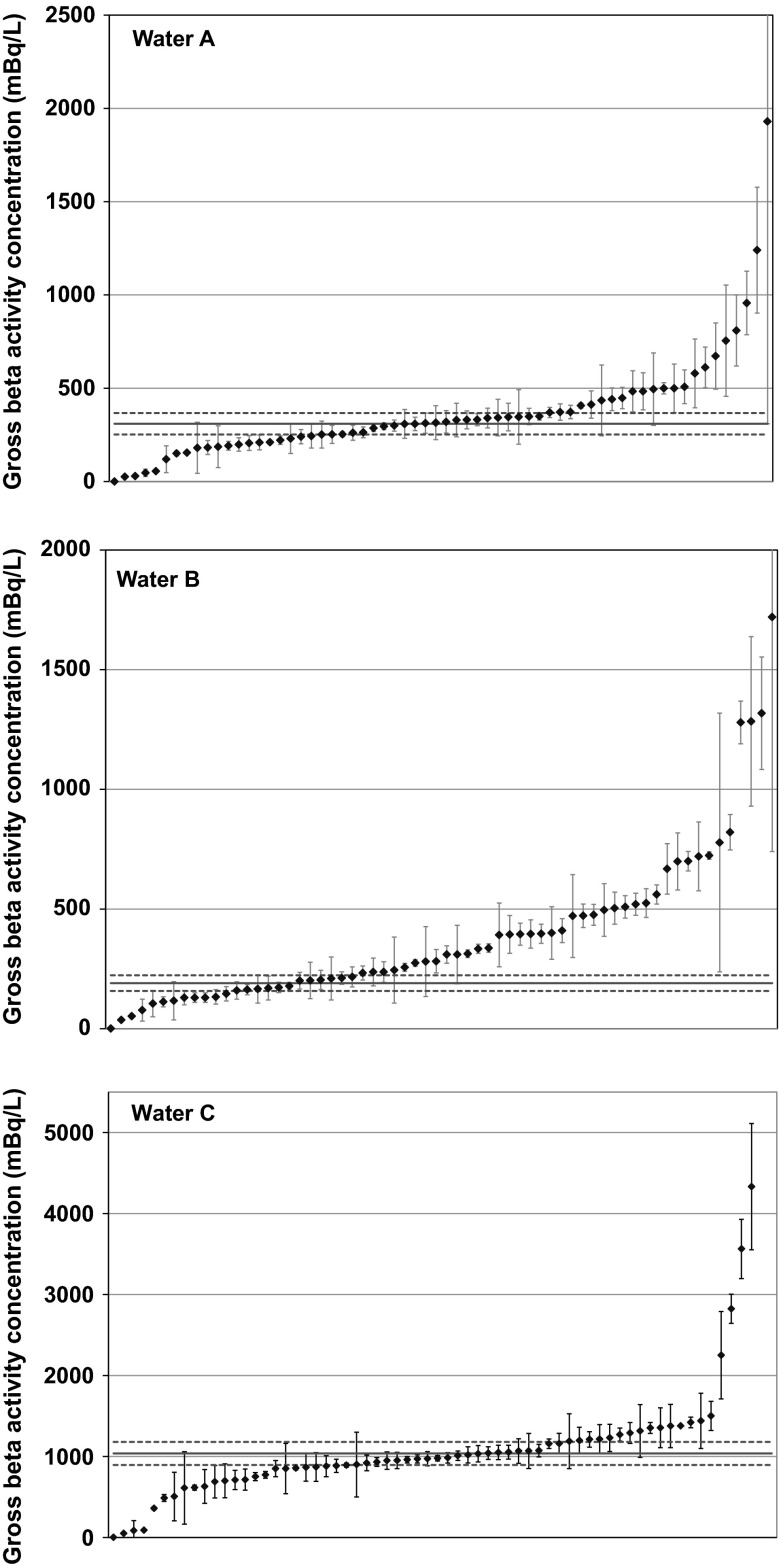
Results of gross beta activity concentration sorted in ascending order. *Error bars* represent expanded uncertainties (*k* = 2)

Only 44 laboratories reported results for the parameter gross alpha activity concentration in sample Water A, while for the same parameter in sample Water B, 70 results were reported. The reported results of gross alpha and beta activity concentration are sorted in ascending order in Figs. 1 and 2. The error bars represent expanded uncertainties (*k* = 2) and solid red line represents the reference value, while dashed red lines represent the corresponding expanded uncertainties. For the better visibility of the data points, the identifier laboratory codes are not indicated on the plots.

Many of the participants’ results deviate by more than two orders of magnitude from the reference values regardless of the techniques used. It is interesting to evaluate the ratio of maximum to minimum reported gross activities (Table 7) and the percentage of compatible results (Table 8). The number of compatible results together with the number of laboratories and their identification codes are presented in Table 9.

**Table 7:** Ratio of the reported maximum to minimum gross activities

Parameter	*A*_max_/*A*_min_		
	Water A	Water B	Water C
Gross alpha activity	1,017	346	93
Gross beta activity	3,050	2,080	3,150

**Table 8:** Percentage of the reported results within ±30 % from the reference value

Parameter	Results within ±30 % deviation (%)		
	Water A	Water B	Water C
Gross alpha activity	36	39	63
Gross beta activity	45	27	61

**Table 9:** Number of laboratories and their ILC identification codes versus the number of reported compatible results

Number of compatible results	Number of laboratories	Laboratory code
6	1	33
5	1	54
4	7	17, 18, 22, 34, 36, 41, 48
3	11	1, 2, 5, 13, 21, 25, 30, 51, 57, 62, 71
2	20	3, 6, 8, 10, 11, 15, 23, 24, 26, 27, 35, 37, 40, 46, 47, 52, 63, 64, 66, 68
1	20	4, 7, 9, 12, 14, 16, 19, 28, 29, 32, 39, 49, 50, 55, 59, 60, 65, 67, 72, 73
0	13	20, 31, 38, 42, 43, 44, 45, 53, 56, 58, 61, 69, 70

**Fig. 3:**
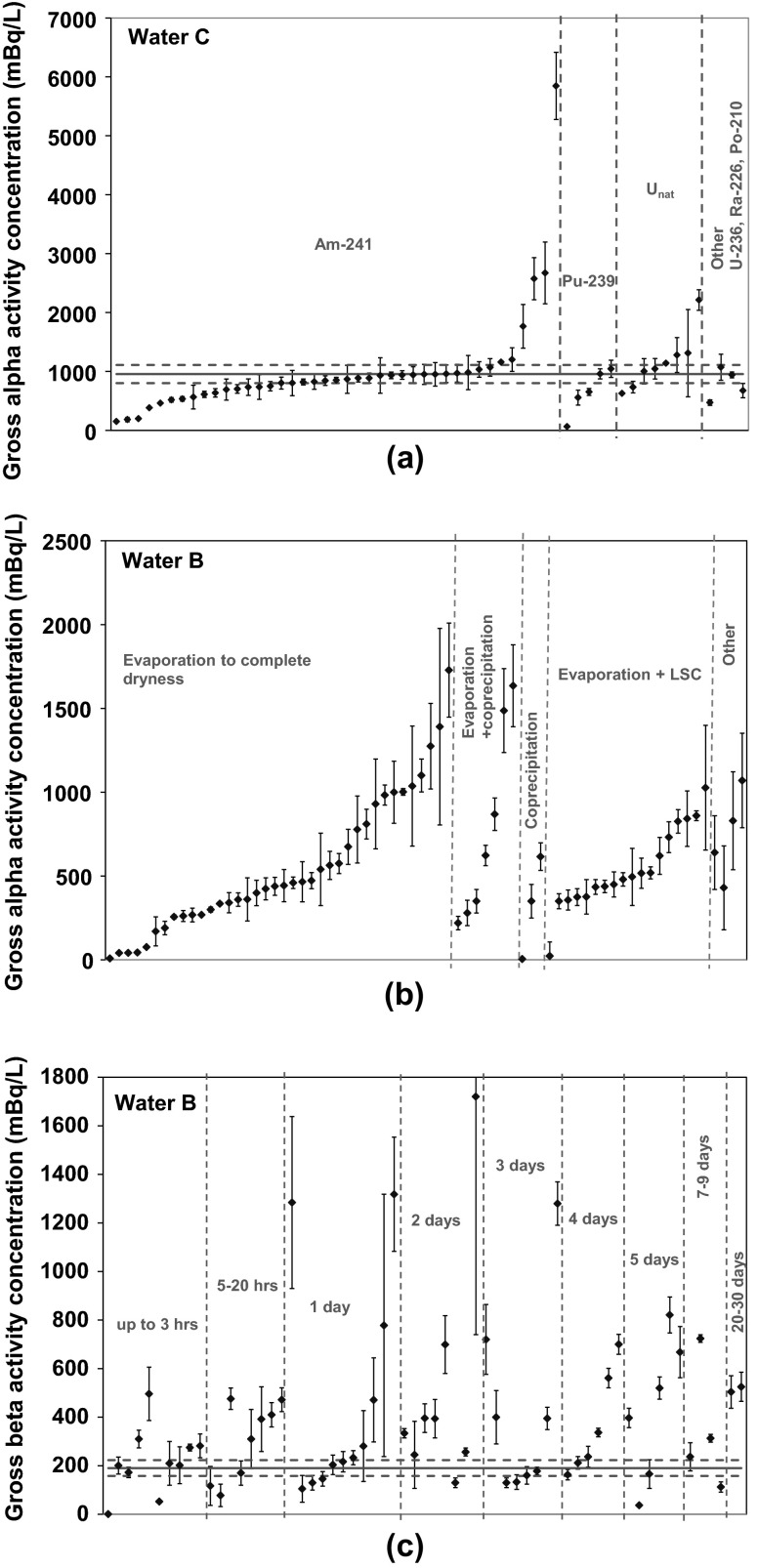
Results sorted on the basis of **a** measurement techniques, **b** sample preparation used and **c** time delay between sample preparation and measurement

**Fig. 4:**
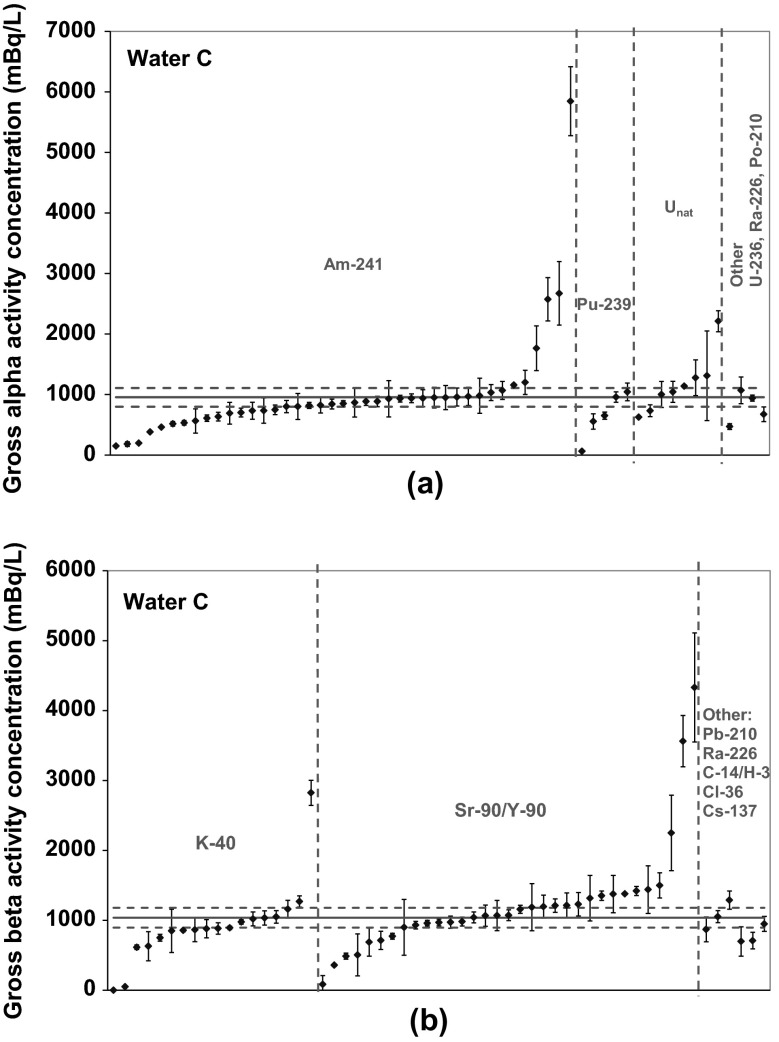
Results sorted on the basis of the radionuclides used for **a** alpha and **b** beta counting efficiency calibration

As shown in Table 9, only 20 laboratories (27 %) out of 71 reported at least half of the results within the reference range. Furthermore, 13 laboratories (18 %) did not report compatible result at all. Among the 20 best performing laboratories we did not find any of the methods to be superior to the other methods. Of these 20 laboratories, only four laboratories used solid scintillation counting and the others applied LSC or proportional counting. During the evaluation of the ILC results, they were sorted by counting technique, sample preparation, radionuclides used for calibration and the time delay between sample preparation and counting. Some of the evaluations are given in graphical form in Figs. 3 and 4.

Comparing the different groups of sorted results, no significant differences between those groups are observed. However, for some groups the available data are limited (e.g. for group “Other”). It is worth to mention that laboratories using the same radionuclide for calibration, as was added as spike in Water C, did not perform better than the laboratories using other radionuclides. All details on the ILC results will be published in the future.

## Conclusions

As mentioned above, only 27 % of the labs reported at least half of the results within the reference range, while 18 % reported incompatible results only. None of the methods was proven to be superior to the others. Even application of the same method in different laboratories does not guarantee comparable results.

The present situation is far from satisfactory knowing that these screening methods are very likely to be used for testing drinking water as foreseen by the drinking water directive [2] and will lead to different decisions seen the large spread in the data. The large spread of the results may be due to influencing factors during both the sample preparation and the measurement process [10, 11]. These influences cannot generally be predicted and it is already difficult to define the measurand for gross activity analysis since the radionuclide composition of the sample is a priory not known.

Additionally, the activity of the sample may substantially change with time as some radionuclides decay and others grow in during the measurement time. For drinking water although a few decay processes are very likely to occur and should be accounted for in the measurement process.

For these reasons, revision of the gross methods is needed [11]. We recommend following strictly accepted common procedures for sample preparation and measurement, to be aware of all decay processes that may affect the measurement, to test procedures for robustness and to set up realistic uncertainty budgets.

The outcome of the analysis may be influenced by the proficiency and training of laboratory personnel too. At least in two European countries (Austria, Switzerland) no gross methods are used for drinking water qualification due to their drawbacks and unreliability. As long as gross activity parameters are included in the European drinking water directive, this interlaboratory comparison should be repeated with pre-defined guideline procedures to be followed.
